# Optically tunable split-ring resonators controlled lead sulfide quantum dots modulator for wide THz radiation

**DOI:** 10.1515/nanoph-2021-0808

**Published:** 2022-03-31

**Authors:** Yifei Xu, Qi Song, Enen Li, Min Zhang, Zhenhua Sun, Tianwu Wang, Fang Liu, Peiguang Yan

**Affiliations:** College of Physics and Optoelectronic Engineering, Shenzhen University, Shenzhen 518060, China; Aerospace Information Research Institute, Chinese Academy of Sciences, Beijing 100094, China; GBA branch of Aerospace Information Research Institute, Chinese Academy of Sciences, Guangzhou 510700, China; Beijing Key Laboratory of Passive Safety Technology for Nuclear Energy, School of Nuclear Science and Engineering, North China Electric Power University, Beijing 102206, China

**Keywords:** optically tunable, PbS quantum dots, split-ring resonators, THz modulator

## Abstract

It is particularly appealing for efficient active terahertz (THz) modulators using photonic structures to enhance light–matter interaction. Here, an optical controlled THz modulator is proposed that combines lead sulfide (PbS) quantum dots with subwavelength metallic split-ring resonators (SRRs) for providing field enhancement. The modulation depth reaches 60.3%, which is approximately 3 times larger than the PbS quantum dots film without SRRs (as reference) in the frequency range of 0.1–1.1 THz. Such significant enhanced THz modulation is mainly due to the local THz field enhancement caused by the SRRs, which is consistent with the simulation result.

## 1 Introduction

THz active modulators, adopted to manipulate the properties of THz propagation, have been investigated as a hot issue due to the enormous applications in the fields of 6G communications and THz imaging [1, 2]. Different active fields and devices have been reported based on optical field, electric field, magnetic field, and thermal field for THz modulation [3], [4], [5], [6], [7]. Taking the advantages of the modulated depth, modulated rate and simple method, optically tunable THz modulator is an attractive choice among all types. The optical controlled modulator is accomplished by incident a laser beam on the semiconductor with the photonic energy exceeding the bandgap, which simultaneously overlaps by THz beam [8, 9].

Previous works have demonstrated that traditional semiconductors, perovskite quantum dots, graphene, transition metal dichalcogenides (TMDCs), and black phosphorus [10], [11], [12], [13], [14] were used to construct optical controlled THz modulator. Traditional semiconductors such as silicon and gallium arsenide (GaAs) always exhibit lower modulation depth because of the reduction recombination of carriers in materials [15, 16]. Due to the stability and weak absorption of light, graphene, black phosphors and TMDCs are limited to a certain extent as materials for the optical modulators. Recently, semiconductor colloidal quantum dots (QDs) have been intensively studied and used in optoelectronic devices on account of their outstanding properties, such as tunability bandgap in a large energy range, high light absorption coefficients and low cost [17, 18]. As a photoelectric semiconductor among the IV–VI group, lead sulfide (PbS) QDs with small optical bandgap and strong quantum confinement make it attractive as the laser medium, photodetectors, and field-effect transistors [19, 20]. The nonlinear optical absorption and photoluminescence in PbS nanocrystals have been reported [21, 22]. Meanwhile, different PbS two-dimensional nanostructures also offer a route for manipulations of THz wave transmission due to the efficient charge generation and stability [23], [24], [25]. Recently, quantum dots were reported to form the optically controlled THz modulator [12, 26]. However, these reports exhibit the low modulation depth and a rather narrow modulation frequency band due to the minimal light–matter interaction length. Therefore, THz modulators with larger modulation depth and wider frequency range are still urgently needed.

Besides, the combination of photonic structures that are thinner than the wavelength of light with nonlinear materials is of particular appealing for THz devices. Integrating subwavelength-scale metallic structure with semiconductors or phase change materials have been a unique platform to manipulate the THz waves and have been used to demonstrate several novel phenomena such as resonance modulation, polarization switching, ultrafast wavefront control, superconducting photonic switching, and strong local field enhancement [27], [28], [29], [30], [31], [32]. Typical subwavelength-scale structures that have been used to enhance light–matter interactions include gratings, split-ring resonators, islands, and hole arrays [33], [34], [35], [36], [37]. For example, subwavelength split-ring resonators (SSRs), as one of the classical photonic structures, to enhance local field confinement within the region of the capacitive gaps [38].

In this work, we experimentally demonstrated a novel optical modulation by superposition of SRRs on synthetic semiconductor colloidal PbS QDs film and proved that the SRRs can provide strong field enhancement at 0.1–1.1 THz, thus leading to a strong increase of the THz modulation depth. The SRRs arrays, which served as local resonant THz concentrators, were fabricated by magnetron sputtering deposition (MSD). In addition, the simulated results show that subwavelength gold SRRs are essentially LC circuits, the THz electric field in-gap can be enhanced by more than an order of magnitude. Our results show that the combination of ultrathin quantum dots film and subwavelength structures has great potential as a THz modulator with high modulation depth.

## 2 Characterization and preparation of PbS QDs samples and experimental setup

PbS quantum dots (QDs) were synthesized by the colloidal hot-injection method same as the previous report [39], [40], [41]. Briefly, the PbS QDs solution could be obtained as follows: firstly, a mixture of 0.45 g of PbO, 1.5 mL of oleic acid (OA), and 18 mL of octadecene (ODE) in a 50 mL flask was heated at 100 °C under vacuum while continuously stirring the solution for the formation of lead oleate. After the lead oxide was dissolved, the same reaction was kept at 125 °C to obtain a yellowish solution under argon flow. A sulfur precursor by mixing 0.18 mL of hexamethyldisilathiane and 10 mL of ODE was quickly injected into the flask at 125 °C. Cool the solution to room temperature. The QDs were then precipitated with acetone and cleaned three times by successive redispersion and precipitation in hexane/ethanol, toluene/acetone and toluene/methanol. They are finally dispersed in octane. Octane solution with PbS QDs doping concentrations of 25 mg/mL was spin-coated onto the Si wafer which had 425 μm thickness and 5 mm × 5 mm size.

We fabricated the SRRs structure by putting a split-ring mask on the QDs layer and the Au split-ring array was obtained by the MSD method. The mask is composed of periodic split-ring with four different directions and the external diameter and internal diameter of the ring are 180 μm and 60 μm, respectively. The Au layer was prepared by the following steps. At first, the air pressure was reduced to 9 × 10^−4^ Pa and injects argon into the cavity. The Au target is coated using DC drives mode. After that, the argon flow rate, current, and duration time were set to be 15 SCCM, 0.2 A, and 90 s. Finally, the sample was taken out when the air pressure returned to 1.0 × 10^5^ Pa.

**Figure 1:**
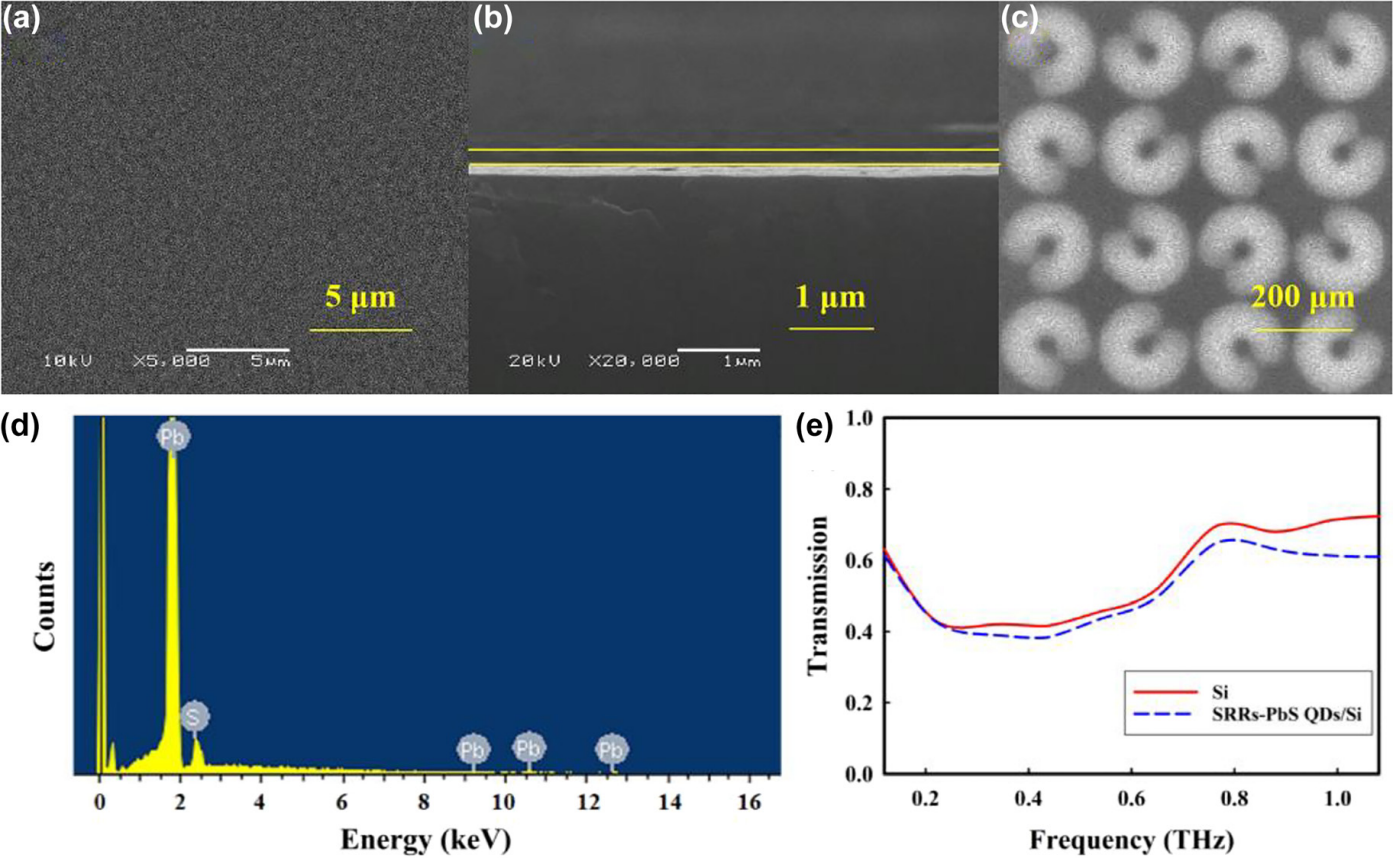
SEM photographs of surface morphology. (a) cross section (b) of the PbS QDs film and (c) the SRRs structure. (d) The EDS spectra detected from an area of the PbS QDs film. (e) The transmission of our device.

Figure 1(a) and (b)show the scanning electron microscopy (SEM) images of morphology and cross-section for the layer of PbS QDs, which show the large uniform area and uniform thickness of the sample. The average PbS QDs size in the thin film is estimated to be a few tens nanometers and the film thickness is about 200 nm. The SEM image of a portion of the fabricated metal arrays is shown in Figure 1(c). The result of quantitative analysis from the energy disperse spectroscopy (EDS) spectra of an area of the PbS QDs is shown in Figure (d). Considering Pb and S only, the atomic percentage of Pb and S are 57.89% and 42.11%, respectively, which is almost in agreement with the formulation of QDs solutions. It can be seen in Figure (e) that the device presented good transmission at bandwidth frequency regions from 0.1–1.1 THz.

**Figure 2:**
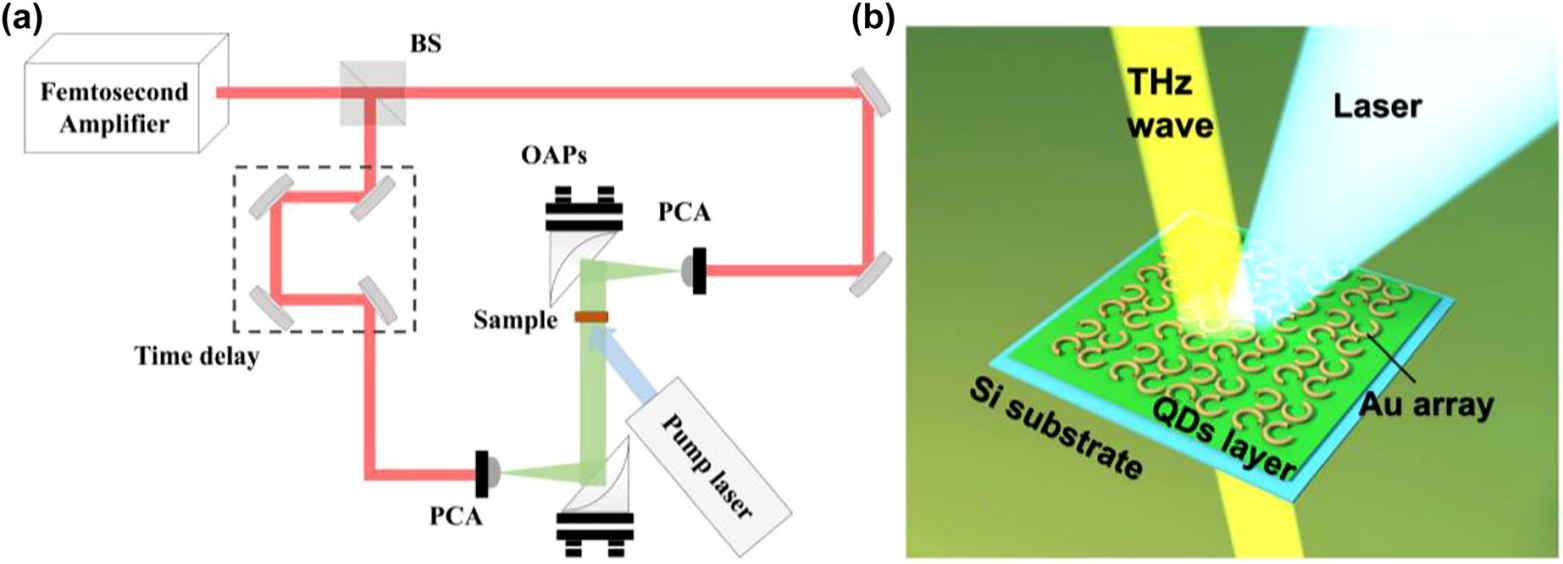
Experimental setup. (a) The schematic diagram of experimental setup of THz-TDS combined with a modulating laser. (b) Designed THz modulator. PCA: photoconductive antenna; OAP: off-axis parabolic.

We utilized the THz time-domain spectroscopy (THz-TDS) to access the THz modulation of the PbS QDs device. The experimental layout was shown in Figure 2(a). Femtosecond pulses (95 fs pulse width, 780 nm center wavelength, 87 MHz repetition rate) from an amplifier were divided into two portions for the generation and sampling. The THz pulse was generated via the femtosecond laser pulse incident on the photoconductive antenna (Batop PCA-40-05-10-800c) while using a pulse generator and radiated by a hemispherical silicon lens at the back of the PCA. The generated THz wave was collimated by an off-axis parabolic (OAP) mirror onto the sample and then the diverging THz radiation was collimated and focused by another OAP onto a PCA for detection. A 980 nm continuous wave laser was mounted to excite the sample with a spot diameter of 5 mm which can overlap the THz beam. All the experiments were performed under a dry purge at room temperature.

## 3 Results and discussion

**Figure 3:**
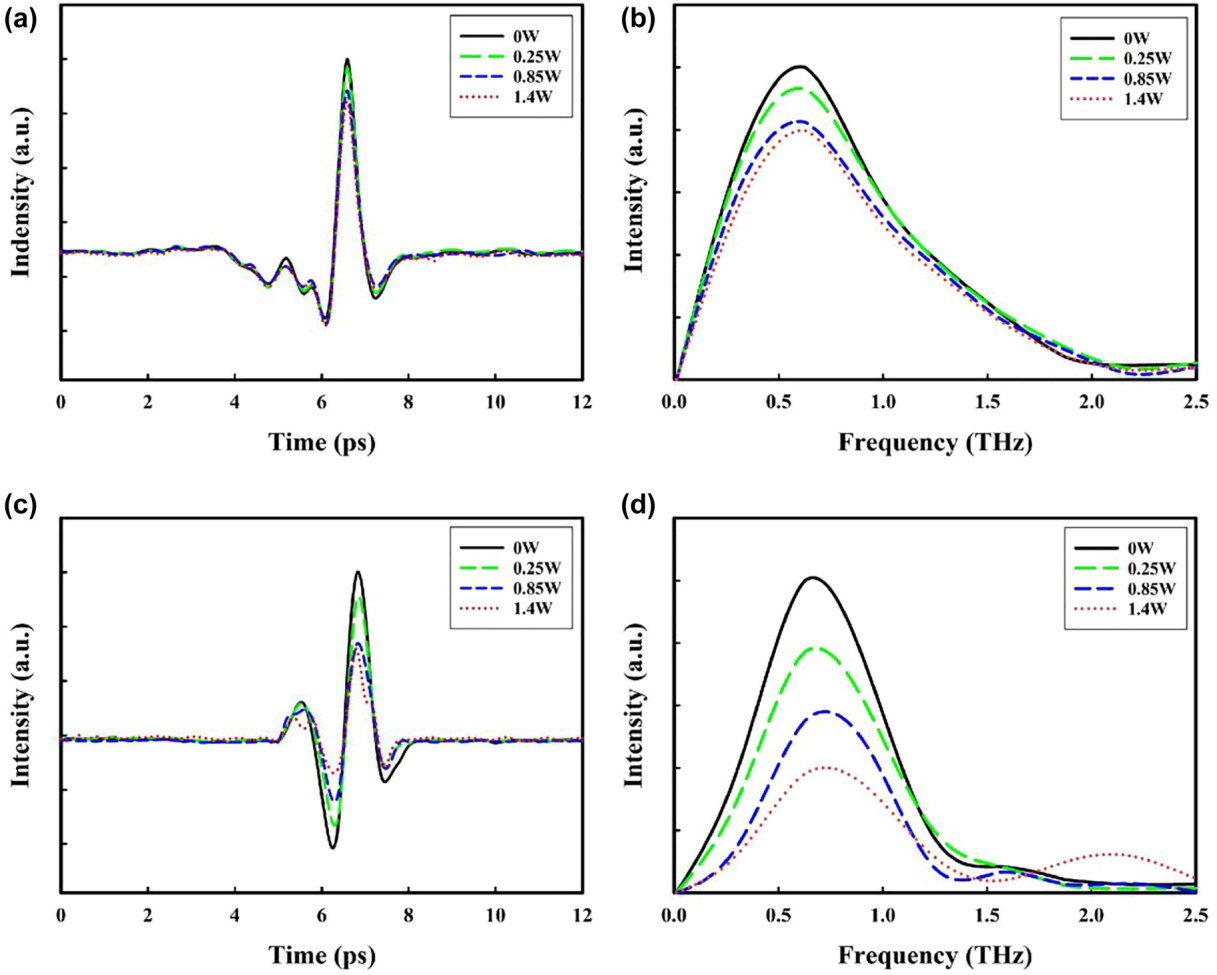
Transmitted THz electric fields of the devices. (a) without SRRs and (c) with SRRs, modulated by different pump powers. (b) and (d) the THz spectra corresponding to (a) and (c), respectively.

Figure 3(b) and (d) show the experimentally measured amplitude spectrums in the THz transmission by various pumping laser powers of two samples, both consisting of PbS QDs film on Si substrate, sample A has gold SRRs on top of the PbS QDs film, while sample B not. Figure 3(a) and (c) display photoinduced modulated electric signals. As one would expect, higher pump intensities induce a larger change in the transparency of the sample due to the photoexcitation of the sample. To investigate the optically-introduced modulation in detail, the modulation depth (MD) can be expressed as [42]:

(1) MD=|T−PT0|T0

**Figure 4:**
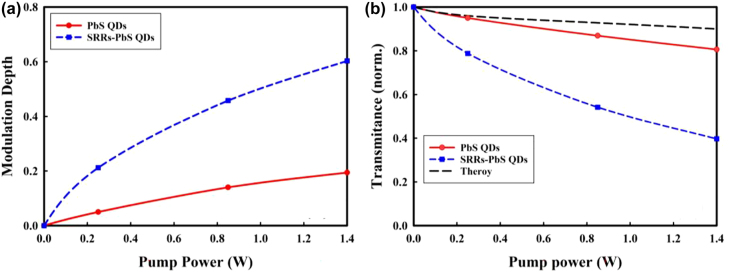
Optical modulation results. (a) MD and (b) normalized THz transmittance for devices with and without SRRs as a function of the pump power.

Here, *T*_P_ and *T*_0_ represent the THz amplitudes after transmitting through samples with and without pumping laser, respectively. The MD as a function of modulated laser for different pump powers is presented in Figure 4(a), it can be noted that the maximum average MD of the sample consisting of PbS QDs and SRRs can reach 60.3% at a pump laser power of 1.4 W while the sample B just 19.4%. The modulation will always be small without metamaterial because there is no SRR resonance to modulate. The obtained results testified that our approach of using SRRs to enhance nonlinear response works. It can be seen that both two samples presented obvious modulation effects at wide-band frequency regions from 0.1 THz to 1.1 THz.

When the PbS QDs (bandgap of ∼1.2 eV [43]) device was excited by a pump laser at 980 nm, the photoexcited electrons and holes could be generated and most of them were then attained an equilibrium due to the electron-hole drift-diffusion. Higher concentration and faster mobility of photo-induced carriers can be achieved under a higher pump laser power, which results in a change in the conductivity of the sample, subsequently, an increase in the THz losses. However, the interaction between THz electric field and PbS QDs is limited because the thickness of PbS QDs is much smaller than the wavelength of the THz wave. For sample A, the comparative experiments indicated that the SRRs structure provides field enhancement, thus leading to a strong increase of the THz nonlinear light–matter interaction. In addition, lower transmission appears in 0.1–0.4 THz due to the THz field confinements of SRRs, it can be noted that the SRRs structure does not absorb THz wave and the transmitted frequencies become higher which demonstrated that sample A could be employed as an efficient modulator.

As shown in Figure 4(b), higher pumping power led to lower THz transmittance for both samples. Here we extract the transmittance coefficient of the PbS QDs using [13, 44]

(2) $$T\left(E\right)=\frac{1}{{(1+\frac{1}{2}\alpha \pi \sigma \left(E\right))}^{2}}$$

Where α≈1/137 is the fine structure constant. Ignoring the phonon effect and other mechanical effects, the nonlinear conductivity up to the third order nonlinear process can be expressed by Floquet expansion [14, 45]:

(3) $$\sigma \left(E\right)=\sqrt{{\left(1+\sigma_{3}\left(E\right)H_{2}\right)}^{2}+\sigma_{3}^{2}\left(E\right)H_{1}^{2}}$$

$\sigma_{3}\left(E\right)=\frac{e^{2}v_{F}^{2}E_{0}^{2}}{\hbar^{2}\omega^{4}}$ , $H_{1}=\frac{13}{48}N\left(\frac{1}{2}\right)-\frac{2}{3}N\left(1\right)+\frac{45}{48}N\left(\frac{3}{2}\right)$ , $H_{2}=2N\left(1\right)$ , $N\left(x\right)=\tanh\left(\frac{x\hbar \omega }{2k_{B}T}\right)$ , *e* is the charge of an electron, $v_{F}\approx 3\times 10^{5}m/s$ is the Fermi velocity [46, 47], *E*_0_ is the strength of the incident field, *ω* is the frequency of the incident photons and T≈300  K is the temperature of the system. The third order conductivity of the PbS QDs could be obtained by [42]:

(4) $$\sigma_{3}\left(P\right)=\frac{e^{2}v_{F}^{2}}{\hbar^{2}\omega^{4}}\frac{2\;\ln\left(2\right)}{c\varepsilon_{0}\pi r^{2}}P$$

*r* is the approximate radius of the incident laser beam and *P* is the power of pumping laser. In Figure 4(b), the theoretical result based on Eq. (2) is close to the measurement result of the sample without SRRs.

To analyze the carrier dynamics in the modulators, we considered that samples were prepared uniformly, the THz direct current (DC) conductivity of PbS QDs layer was theoretically calculated by using a simple model based on the transmission of the far-infrared amplitude *T* through a thin Drude metal film on a semi-infinite insulating substrate [48]:

(5) $$\sigma_{\mathrm{DC}}=\frac{1+n_{s}}{Z_{0}d_{f}}\left(\frac{1}{1+\Delta T(t)/T_{0}}-1\right)$$

**Figure 5:**
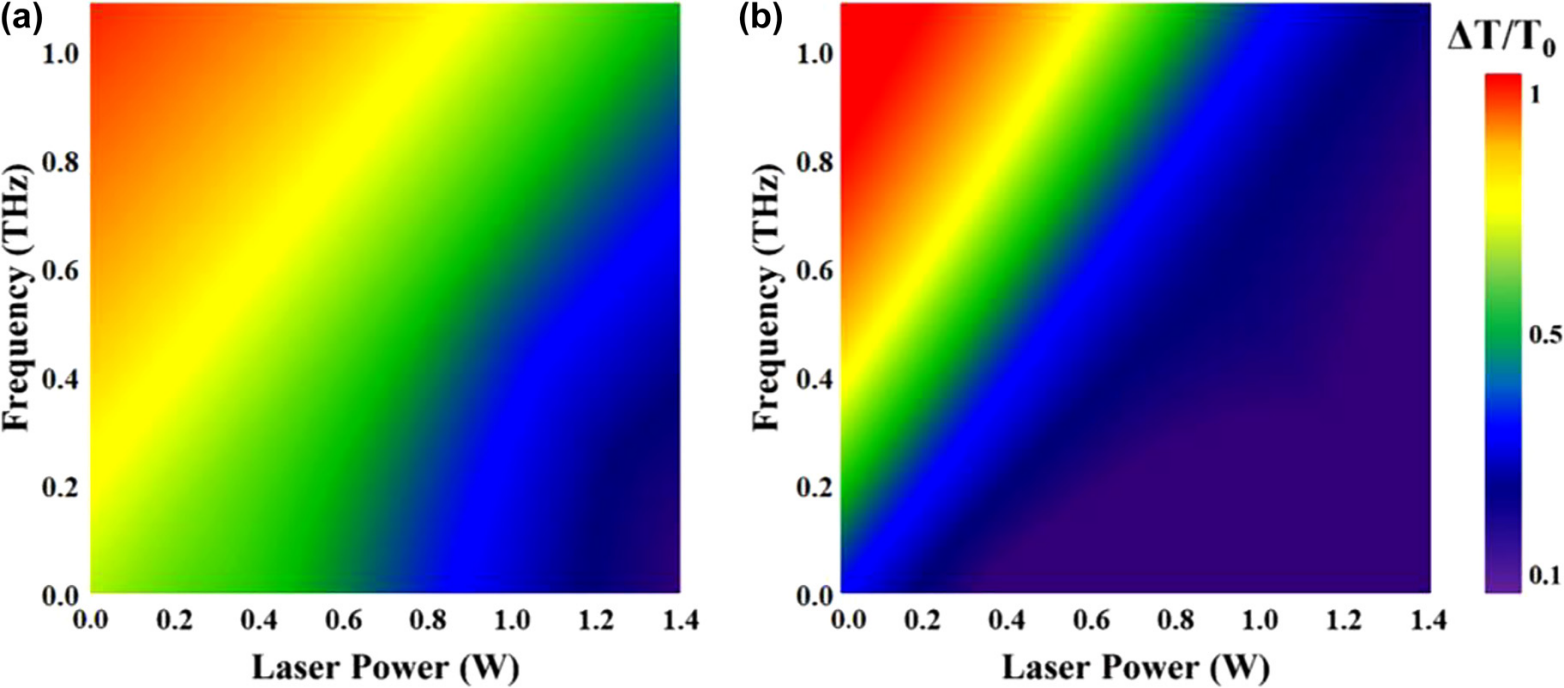
The frequency resolved transmission change spectra under different pump powers. (a) without SRRs and (b) with SRRs.

**Figure 6:**
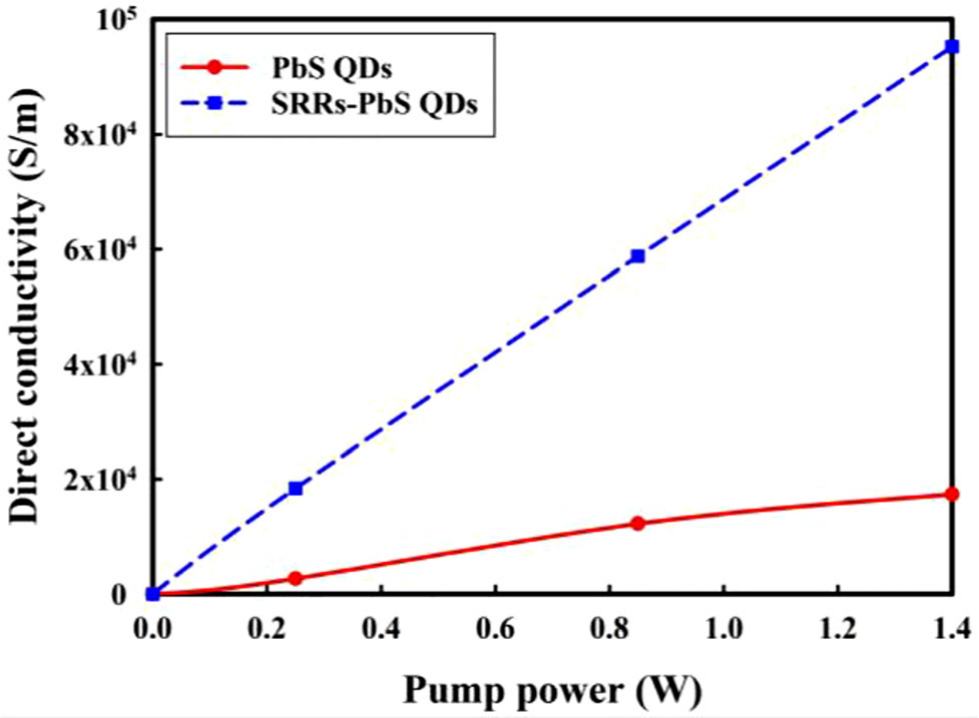
Pump power dependent dc conductivity obtained from Eq. (5).

Where *n*_s_ is the refractive index of Si substrate, *Z*_0_ = 377 Ω is the impedance of free space, and *d*_f_ is the thickness of PbS QDs layer, ∆*T* = *T*_p_ − *T*_0_. As shown in Figure 5(a) and (b), the frequency-resolved transmission change ∆*T*/*T*_0_ is closely related to pump power for both two samples. The maximum transmission change of the sample with SRRs under 1.4 W pump power is over 2 times higher than that of the sample without SRRs. Figure 6 shows that there is a linear relationship between DC conductivity and the pump power from the experimental data using Eq. (5). Large conductivity enhancement occurs in the sample with SRRs.

**Figure 7:**
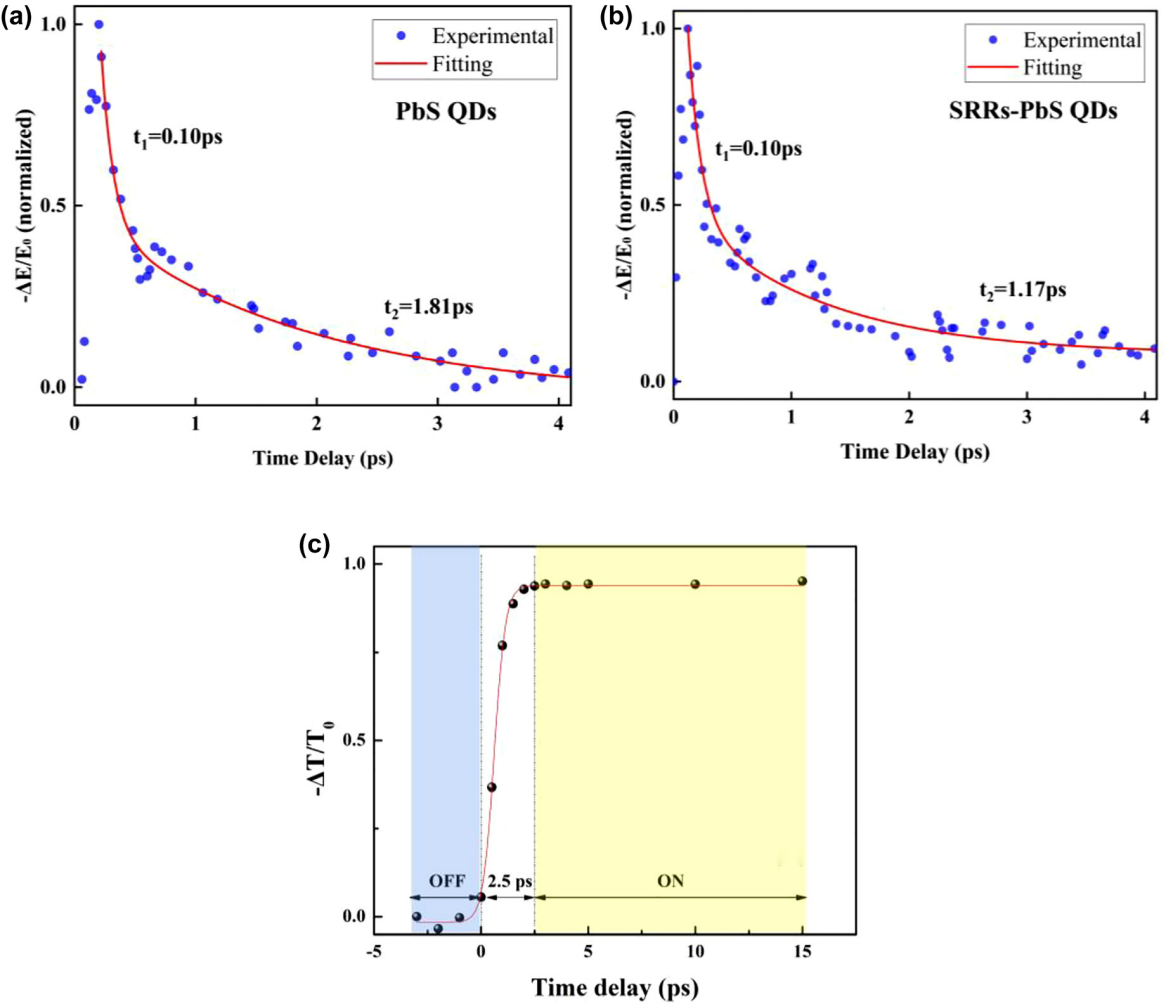
OPTP results and response time. (a) and (b) normalized charge carrier dynamics in the PbS QDs device without and with SRRs, respectively, measured using the OPTP system at excitation pump fluence 31 μJ mm^−2^. (c). Ultrafast response time of the device with SRRs.

Figure 7(a) and (b) show the normalized charge carrier dynamics in the PbS QDs device without and with SRRs, respectively, measured using the OPTP system. A Ti: sapphire femtosecond laser with a central wavelength of 800 nm is used as the laser source for pump detection. The pump energy density is 31 μJ/mm^2^. The probe trace exhibits a rapid rise in the photoconductivity and is followed by a biexponential relaxation as follows:

(6) $$-\frac{\Delta E}{E_{0}}=A_{1}\;\exp\left(-\frac{\tau }{t_{1}}\right)+A_{2}\;\exp\left(-\frac{\tau }{t_{2}}\right)$$

The relaxation processes of the biexponential decay function can be attributed to the carrier–carrier scattering and the carrier-phonon scattering. *t*_1_ and *t*_2_ reflect the fast and slow relaxation process, respectively. In our experiments, the *t*_1_ and *t*_2_ for PbS QDs are 0.10 ps and 1.81 ps, respectively. The initial photoexcited carrier process in both devices is the same. However, the exciting dynamics can be recognized different, *t*_1_ value of the SRRs device is 0.1 ps, while *t*_2_ value is 1.17 ps, which could be attributed to the defects and the SRRs structure of the device [14, 49]. Figure 7(c) shows the induced relative change in THz transmission of the devices with SRRs as a function of the pump-probe time delay. Δ*T* = *T* − *T*_0_ is the transmission change, *T* and *T*_0_ are the transmissions of a THz wave through the sample with and without optical excitation, respectively. The rise of *−*Δ*T*/*T*_0_ can be attributed to an increase in photoconductive free carriers density. The switching time of the device is 2.5 ps could be obtained.

To further understand the surface field enhancement of the SRRs, the numerical simulation is performed. A unit-cell of the proposed THz modulator is considered. It consists of a metallic SSRs structure which is composed of four gold split-rings with different directions and a PbS QDs layer on Si substrate. In this model, the external diameter and internal diameter of the ring are 180 μm and 60 μm respectively. In our interested frequency range of 0.1–1.1 THz, the metallic layers made of 0.2 μm gold can be regarded as approximate perfect electric conductor with conductivity $\sigma =4.56\times 10^{7}\;\;S/m$ . The layer of the PbS QDs has a thickness of 150 nm and its conductivity can be obtained by Eq. (5). Unit cell boundary conditions were imposed in the *x* and *y* directions, and an open boundary condition in the *z*-direction. The unit-cell structure is illuminated by a normal incident THz wave with the E field polarized in the *x*-direction and the H field polarized in the *y*-direction.

**Figure 8:**
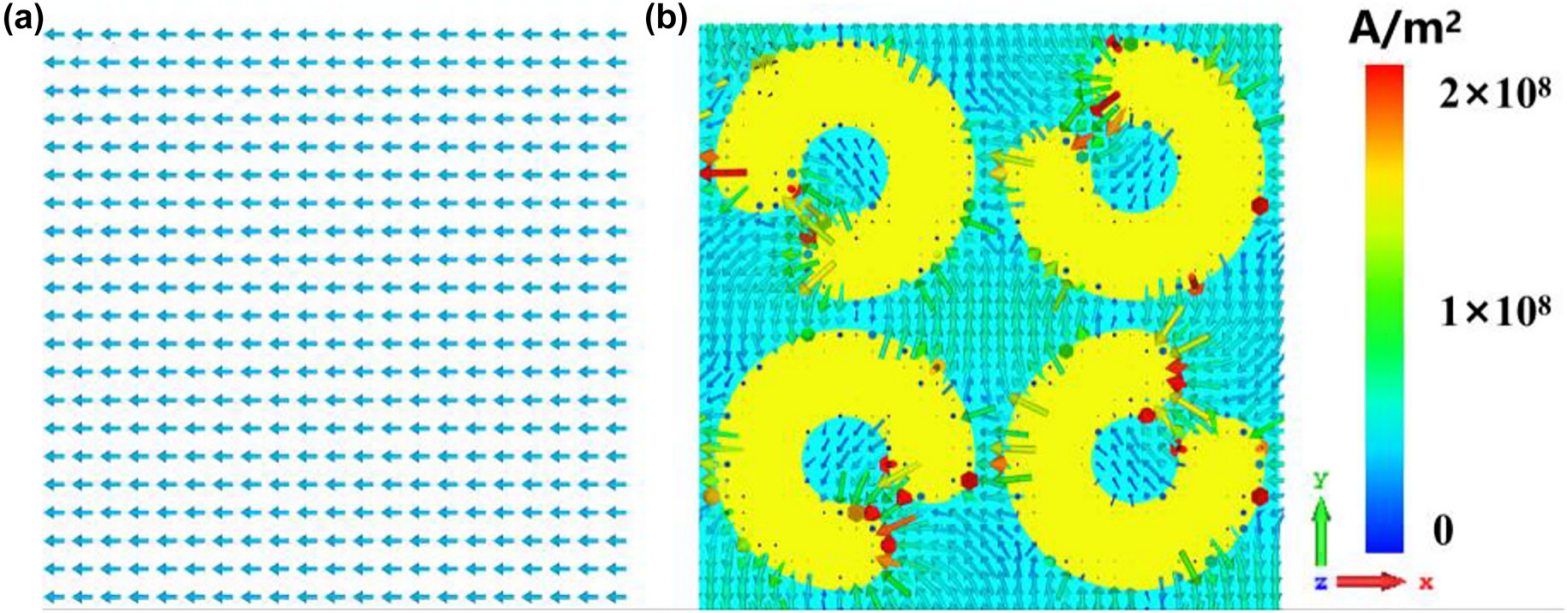
Surface current density distribution. (a) without SRRs and (b) with SRRs at 0.6 THz.

**Figure 9:**
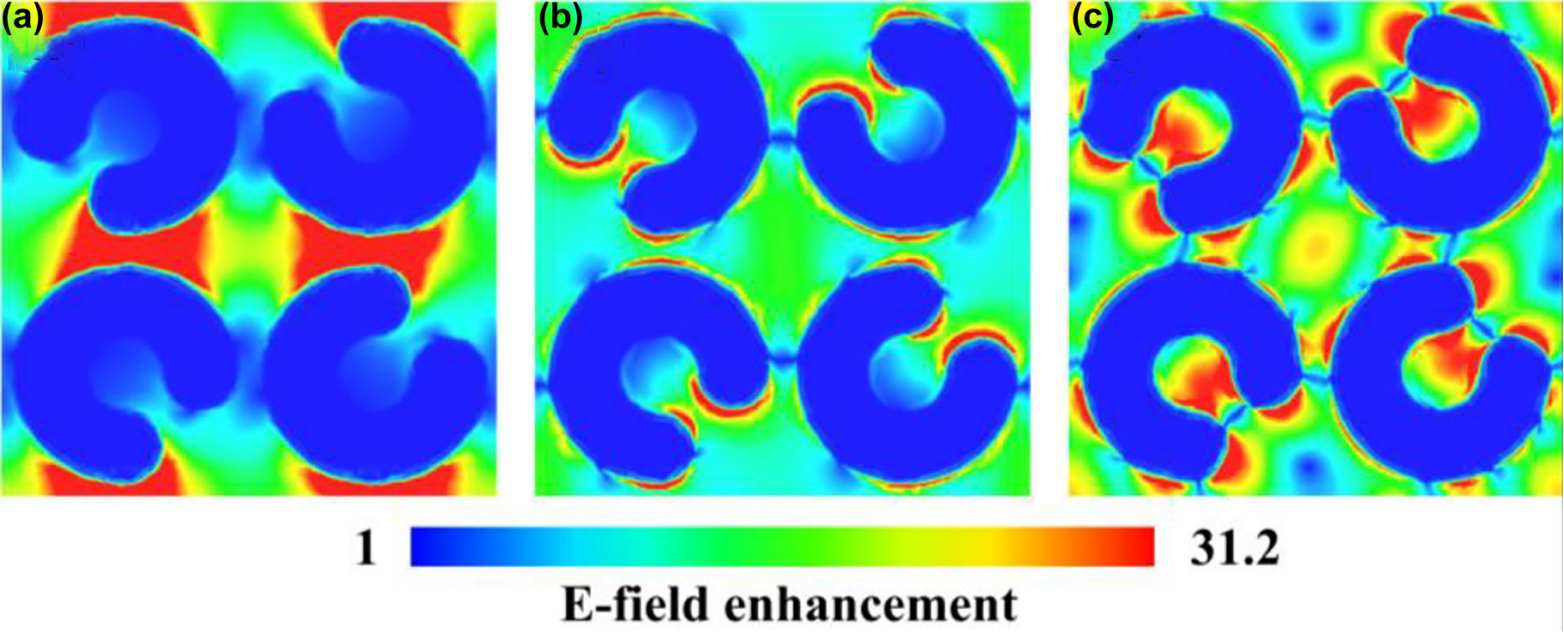
Numerically extracted E-field strengths at different frequencies. (a) 0.1 THz (b) 0.6 THz (c) 1.1 THz showing the E-field enhancement in the resonator gaps.

The distribution of surface current density without and with metallic arrays in the perspective of the x-y plane is shown in Figure 8(a) and (b), respectively. We confirm that the surface current distribution on the split ring of SRRs is circular, which is characteristic to LC resonance, and the current density in-gap is much larger than other areas. The simulations show a trend that is markedly similar to the experimental results, the interaction between THz wave and material is significantly enhanced within the gaps. Figure 9(a)–(c) presents the numerically extracted electric field distributions on the *x*–*y* plane at frequencies 0.1 THz, 0.6 THz, 1.1 THz, implying the designed metallic SRRs structure has different field confinements for different THz frequencies. The E-field in the resonator gaps shows nearly 30 times enhancement.

For comparison, we list the reported optically controlled THz modulators in Table 1. It is noted that our proposed device exhibits the main advantages of the easy integration, wide modulation bandwidth, large modulation depth, and transmission rate. Moreover, this device also has the merit of large area because both the “spin-coating” method and the MSD method permit the PbS QDs film and the SRRs array to achieve the controllable large-size fabrication in a low-cost manner. In brief, our device with its manufacturing technique can be a good candidate for constructing the practical THz system, not only restricted as a modulator.

**Table 1:** Comparison of the optically controlled THz modulators based on different material systems.

Materials	Device type	Modulation depth	Frequency	Transmission rate
GaAs/AlAs [3]	Multiple quantum well	30%	0.2–1 THz	–
ErAs/GaAs [50]	Metamaterial	35%	0.75 THz	0–30%
InSb [51]	Grating	46.7%	1.5 THz	40–60%
TiS_2_ [13]	Nanosheet	30–50%	0.1–1 THz	–
PbS QDs [this work]	Matesurfaces	60.3%	0.1–1.1 THz	40–65%

## 4 Conclusions

In conclusion, an optically tuned wide-band THz modulator based on the SRRs controlled PbS QDs structure was demonstrated. Remarkably, embedding gold SRRs on the thin film of PbS QDs can enhance the modulation depth to 60.3% (in comparison to 19.4% of the bare QDs film) by an external 980 nm laser pump power of 1.4 W. For the PbS QDs devices without and with SRRs, the fast component *t*_1_ are both 0.1 ps, the slow component *t*_2_ values are 1.81 ps and 1.17 ps. Moreover, the switching time of ∼2.5 ps was obtained. The surface current density distribution and the electric field distribution of our device were simulated, yielding well agreement with the experimental results that metallic resonators induced field enhancement significantly increases the interactions of the light material. Our results indicate that the device combining the split-ring resonators with quantum dots can be used as an effective scheme for the THz modulator.

## References

[1] Yang Y. H., Yamagami Y., Yu X. B., Pitchappa P., Singh R. (2020). Terahertz topological photonics for on-chip communication. *Nat. Photonics*.

[2] Jepsen P. U., Cooke D. G., Koch M. (2011). Terahertz spectroscopy and imaging-Modern techniques and applications. *Laser Photon. Rev.*.

[3] Libon I. H., Baumgartner S., Hempel M. (2000). An optically controllable terahertz filter. *Appl. Phys. Lett.*.

[4] Song Q., Chen H., Zhang M. (2021). Broadband electrically controlled bismuth nanofilm THz modulator. *APL Photonics*.

[5] Ishii T., Yamakawa H., Kanaki T. (2019). Large terahertz magnetization response in ferromagnetic nanoparticles. *Appl. Phys. Lett.*.

[6] Chen H. T., Yang H., Singh R. (2010). Tuning the resonance in high temperature superconducting terahertz metamaterials. *Phys. Rev. Lett.*.

[7] Zhang Z., Zhang Y., Luo Y. (2021). Terahertz perfect absorber based on flexible active switching of ultra-broadband and ultra-narrowband. *Opt. Express*.

[8] Rossi F., Kuhn T. (2002). Theory of ultrafast phenomena in photoexcited semiconductors. *Rev. Mod. Phys.*.

[9] Rahm M., Li J. S., Padill W. J. (2013). THz wave modulators: a brief review on different modulation techniques. *J. Infrared. Millimeter Terahertz Waves*.

[10] Alius H., Dodel G. (1991). Amplitude-, phase-, and frequency modulation of far-infrared radiation by optical excitation of silicon. *Infrared. Phys.*.

[11] Vogel T., Dodel G., Holzhauer E., Salzmann H., Theurer A. (1992). High-speed switching of far-infrared radiation by photoionization in a semiconductor. *Appl. Opt.*.

[12] Li S. H., Li J. S. (2018). Terahertz modulator a using CsPbBr_3_ perovskite quantum dots heterostructure. *Appl. Phys. B*.

[13] Wen Q. Y., Wei T., Mao Q (2014). Graphene based all-optical spatial terahertz modulator. *Sci. Rep.*.

[14] Song Q., Chai L., Chen J. Q., Liu W. N., Hu M. L. (2021). Optically tuned wide-band terahertz modulation, charge carrier dynamics and photoconductivity of femtosecond laser ablated titanium disulfide nanosheet devices. *IEEE. J. Sel. Top. Quant.*.

[15] Nozokido T., Minamide H., Mizuno K. (1995). Generation of submillimeter wave short pulses and their measurements. *Riken Rev.*.

[16] Nozokido T., Minamide H., Mizuno K. (1997). Modulation of submillimeter wave radiation by laser produced free carriers in semiconductors. *Electron. Commun. Jpn. Pt. II.*.

[17] Tang J., Liu H., Zhitomirsky D. (2012). Quantum junction solar cells. *Nano Lett.*.

[18] Chuang C. H. M., Brown P. R., Bulovic V., Bawendi M. G. (2014). Improved performance and stability in quantum dot solar cells through band alignment engineering. *Nat. Mater.*.

[19] Wang H., Kubo T., Nakazaki J., Kinoshita T., Segawa H. (2013). PbS-quantum-dot-based heterojunction solar cells utilizing ZnO nanowires for high external quantum efficiency in the near-infrared region. *J. Phys. Chem. Lett.*.

[20] Malyarevich A. M., Gaponenko M. S., Yumashev K. V. (2006). Nonlinear spectroscopy of PbS quantum-dot-doped glasses as saturable absorbers for the mode locking of solid-state lasers. *J. Appl. Phys.*.

[21] Konstantatos G., Howard I., Fischer A. (2006). Ultrasensitive solution-cast quantum dot photodetectors. *Nature*.

[22] Dogan S., Bielewicz T., Cai Y., Klinke C. (2012). Field-effect transistors made of individual colloidal PbS nanosheets. *Appl. Phys. Lett.*.

[23] Wang H., Wu G., Qiu J., Dong G. (2015). Direct evidence on the energy transfer of near-infrared emission in PbS quantum dot-doped glass. *Opt. Express*.

[24] Skurlov I. D., Ponomareva E. A., Ismagilov A. O. (2020). Size dependence of the resonant third-order nonlinear refraction of colloidal PbS quantum dots. *Photonics*.

[25] Lauth J., Failla M., Klein E., Klinke C., Kinge S., Siebbeles L. (2019). Photoexcitation of PbS nanosheets leads to highly mobile charge carriers and stable excitons. *Nanoscale*.

[26] Yang Y., Li J. N., Liu H. C. (2020). Enhanced frequency and amplitude modulation of THz metasurfaces based on CdSe/CdS quantum rods. *Opt. Commun.*.

[27] Kumar A., Solanki A., Manjappa M. (2020). Excitons in 2D perovskites for ultrafast terahertz photonic devices. *Sci. Adv.*.

[28] Manjappa M., Solanki A., Kumar A., Sum T. C., Singh R. (2019). Solution-processed lead iodide for ultrafast all-optical switching of terahertz photonic devices. *Adv. Mater.*.

[29] Tan T. C., Srivastava Y. K., Ako R. T. (2021). Active control of nanodielectric-induced THz quasi-BIC in flexible metasurfaces: a platform for modulation and sensing. *Adv. Mater.*.

[30] Srivastava Y. K., Manjappa M., Cong L. Q. (2018). A superconducting dual-channel photonic switch. *Adv. Mater.*.

[31] Singh R., Zheludev N. (2014). Materials superconductor photonics. *Nat. Photonics*.

[32] Gerislioglu B., Ahmadivand A., Pala N. (2018). Tunable plasmonic toroidal terahertz metamodulator. *Phys. Rev. B*.

[33] Deinert J. C., Iranzo D. A., Perez R., Jia X. (2020). Grating-graphene metamaterial as a platform for terahertz nonlinear photonics. *ACS Nano*.

[34] Cong L., Srivastava Y. K., Solanki A. (2017). Perovskite as a platform for active flexible metaphotonic devices. *ACS Photonics*.

[35] Pitchappa P., Kumar A., Prakash S. (2021). Volatile ultrafast switching at multilevel nonvolatile states of phase change material for active flexible terahertz metadevices. *Adv. Funct. Mater.*.

[36] Ahmadivand A., Gerislioglu B., Ramezani Z. (2019). Gated graphene island-enabled tunable charge transfer plasmon terahertz metamodulator. *Nanoscale*.

[37] Tian Z., Singh R., Han J. G. (2010). Terahertz superconducting plasmonic hole array. *Opt. Lett.*.

[38] Fan K., Hwang H. Y., Liu M., Strikwerda A. C., Averitt R. D. (2013). Nonlinear terahertz metamaterials via field-enhanced carrier dynamics in GaAs. *Phys. Rev. Lett.*.

[39] Hines M. A., Scholes G. D. (2003). Colloidal PbS nanocrystals with size-tunable near-infrared emission: observation of post-synthesis self-narrowing of the particle size distribution. *Adv. Mater.*.

[40] Artem A. B., Stefanie N., Huib J. B., Laurent O. (2013). Charge trapping dynamics in PbS colloidal quantum dot photovoltaic devices. *ACS Nano*.

[41] Sun Z., Sitbon G., Pons T., Bakulin A. A. (2015). Reduced carrier recombination in PbS – CuInS_2_ quantum dot solar cells. *Sci. Rep.*.

[42] Yang D. S., Jiang T., Cheng X. A. (2017). Optically controlled terahertz modulator by liquid-exfoliated multilayer WS_2_ nanosheets. *Opt. Express*.

[43] Sun Z., Liu Z., Li J., Tai G. A., Yan F. (2012). Infrared photodetectors based on CVD-grown graphene and PbS quantum dots with ultrahigh responsivity. *Adv. Mater.*.

[44] Ang Y. S., Sultan S., Zhang C. (2011). Nonlinear optical spectrum of bilayer graphene in the terahertz regime. *Appl. Phys. Lett.*.

[45] Varma S. J., Kumar J., Liu Y., Layne K., Ajayan P. M. (2017). 2D TiS_2_ layers: a superior nonlinear optical limiting material. *Adv. Opt. Mater.*.

[46] Majumder F. A., Swoboda H. E., Kempf K., Klingshirn C. (1985). Electron-hole plasma expansion in the direct-band-gap semiconductors CdS and CdSe. *Phys. Rev. B Condens. Matter*.

[47] Shchekin O. B., Deppe D. G., Lu D. (2001). Fermi-level effect on the interdiffusion of InAs and InGaAs quantum dots. *Appl. Phys. Lett.*.

[48] Lui K. P. H., Hegmann F. A. (2001). Ultrafast carrier relaxation in radiation-damaged silicon on sapphire studied by optical-pump-terahertz-probe experiments. *Appl. Phys. Lett.*.

[49] Wen X. L., Manjappa M., Srivastava Y. K. (2018). Ultrafast all-optical switching of germanium-based flexible metaphotonic devices. *Adv. Mater.*.

[50] Chen H. T., Padilla W. J., Zide J. M., Bank S. R., Averitt R. D. (2007). Ultrafast optical switching of terahertz metamaterials fabricated on ErAs/GaAs nanoisland superlattices. *Opt. Lett.*.

[51] Deng L. Y., Teng J. H., Liu H. W., Wu Q. Y., Tang J. (2013). Direct optical tuning of the terahertz plasmonic response of InSb subwavelength gratings. *Adv. Opt. Mater.*.

